# Patient‐reported outcomes (PROs) versus patient‐reported outcome measures (PROMs)—Is there a difference?

**DOI:** 10.1002/cre2.112

**Published:** 2018-06-19

**Authors:** Asbjørn Jokstad

**Affiliations:** ^1^ Institute of Clinical Dentistry, Faculty of Health Sciences UiT The Arctic University of Norway Norway

We all agree that patient‐reported outcomes (PROs) in clinical research are important and relevant and that investigators should strive to incorporate patient experiences in future trials. I recently witnessed a verbal encounter where several colleagues reiterated very passionately that we needed to adopt the term patient‐reported outcome measure (PROM) as a more proper term than simply PRO. Being a non‐native English speaker, I struggle to reconcile that PROs and PROMs can be synonyms, and I fail to understand why colleagues and Wikipedia declare that “the term PROs is becoming increasingly synonymous with ‘patient‐reported outcome measures’ (PROMs)” (Wikipedia, 2018). A few attempts to solicit a logical rationalization have been unsuccessful. Hence, I wonder if not the blend of the two terms are most likely the result of a Humpty‐dumpty approach to semantic definition, which ought to raise some cautious concerns: “When I use a word,” Humpty Dumpty said, in rather a scornful tone, “it means just what I choose it to mean ‐ neither more nor less.” “The question is,” said Alice, “whether you can make words mean so many different things.” “The question is,” said Humpty Dumpty, “which is to be master—that's all.” (Carroll, 1872).

All dentists have throughout decades and centuries used PROs on an individual basis – did your pain disappear after the tooth was restored, sir? Do you find the shade of your new bridge acceptable, madam? Should we hold on for one more week before doing a root filling, or do you want to proceed now, etc.? True, the dichotomy of the PRO examples above reflects the everyday of any clinician, which is that dentists tend to operationalize outcome alternatives according to potential need for an action or inaction. True also, sometimes the clinician obtains a better clinical judgment of the patient's predicament by additional probing questions and perhaps even categorization of, e.g., possible effects of temperature change, diurnal variation, particular stomatognathic functions, and eating spicy/sweet/tough foods. The dialogues are seldom pre‐planned or structured, and are often guided by nonverbal communication or by clues and nuances detected in the patient's answers.

The general idea of constructing a PROM instrument or tool, however, is that a measure based on a given set of questions will present the patients' perception of their functional well‐being and health status for the clinician, or any other stakeholder. For clarification for those who cannot keep up contemporary newspeak, both PROs and PROMs differ from patient‐centered outcomes (PCOs), which denotes surveys issues and concerns that are specific to patients, as well from patient‐reported experience measures (PREMs), which focus on patients' overall experience with care. Generic PROM instruments can compare across different cares or cures, alternatively between care providers, at least in theory. Condition‐specific PROM instruments are constructed more purposely for a particular health condition. Correctly constructed PROM instruments have the potential to generate data that enable comparisons of, e.g., providers' performance, and thereby empowers both patients, policymakers and other stakeholders to debate political issues such as managed care, quality of services, access to care equity, and health resource priorities. The caveat is how one construes a “correctly constructed” PROM instrument.

With all respect to Wikipedia for all their great work, I disagree that PROs and PROMs are synonyms. Both dimensions are highly relevant, notwithstanding that PROs are the focus of the experts in clinical dentistry and mostly applied to individual patient care. In contrast, expertise on theories and techniques of psychological measurements is required to construct reliable and valid PROM instruments. Mention to a dentist, e.g., Cronbach's α or test internal consistency, and he or she will most likely roll their eyes and ask why they need this knowledge to treat patients properly. On the other hand, a PROM instrument is not particularly difficult to construct for any reflective practitioner who has accumulated ample clinical experiences. The first step in a PROM instrument development process is to judiciously compose a set of questions that one anticipate will reflect the patient's perception of their functional well‐being and health status before and after treatment. A simplistic example is whether patient‐reported limitations in intake of hard foods due to poor dentition will change after you have provided the patient with more robust teeth (AKA extensive prosthodontic care). Amongst the approximately 25 systematic reviews reporting on the use of PROs in prosthodontics, only two focus on patient‐reported nutritional changes, (Sanchez‐Ayala, Lagravère, Gonçalves, Lucena & Barbosa, 2010; Yamazaki, Martiniuk, Irie, Sokejima & Lee, 2016) and there are no validated PROM instruments that address nutritional intake and –status, at least not in dentistry. Admittedly, a few PROM instruments come close, such as the Oral Health Impact Profile (OHIP) 49 questions, ‐20 questions, or ‐14 questions versions, but they address principally perceived physical or social disability relative to chewing and eating.

Hence, a first step would be to identify different hard foods that dictate a robust dentition to shear and fragment. Food items you identify are (a) artichokes, (b) baby back‐ribs, (c) bagels, (d) beef jerky, (e) bruschetta, (f) corn on the cob, (g) crispbread, (h) dried cod, (i) green apple, (j) power bars, (k) taco shells, (l) ice‐cubes, (m) liquorice, (n) nuts, (o) raw carrots, or (p) toffee, and the responders are requested to report how often they consume any of these items. One may categorize the answers by, e.g., five categories such as (0) never, (1) hardly ever, (2) occasionally, (3) fairly often, or (4) very often. Thus, your newly developed PROM instrument consists of 16 rows × 5 columns, where the expected PROM can vary between sum scores of 0 and 64. A low sum score indicates that the responder limits their intake of the itemized hard foods. Voila –a new PROM instrument has been constructed! However, the challenge is really to establish whether the instrument is reliable and valid. For a start, why are 16 hard foods included, instead of say, four or six? Why is the score categorized from zero to four relative to time, instead of say, yes‐no or perhaps even by using a visual‐analogue scale (VAS)? More specifically, is it likely that all the itemized hard foods can be consumed by all potential future responders, or are there issues such as allergy (green apple, nuts, raw carrots), health risks (liquorice), availability (artichoke, dried cod) which signify that these items should better be left out in a PROM instrument. To summarize, multiple methodological and statistical challenges arise when attempting to construct a PROM instrument with acceptable reliability, face validity, content validity, construct validity, convergent validity, and discriminant validity.

I agree that both patients, providers and other stakeholders can likely benefit from adopting PROM instruments and perhaps even comparing PROMs. However, we need to be aware that a PROM can also be a misuse as a type of PRO reductionism. There will always be a range between qualitative “soft‐data” based on a sincere and empathetic patient interview and any kind of PROMs.

A promising potential of PROM instruments is to improve patient care. An example in the field of oral and dental medicine is how cancer patients that develop painful oral mucositis (OM) secondary to the cancer therapies are being managed today. In the past, a clinician appraised the severity of OM by scoring the appearance of the OM while today the importance of the patient‐reported symptoms of OM are recognized. Especially at the time when the burden of OM is the greatest, many patients are unable to open their mouth for an intraoral inspection. Under such conditions, it is difficult to define primary outcomes in trials of the effectiveness of interventions to prevent or cure OM. One emerging PROM instruments is PROMS (Patient‐Reported Oral Mucositis Symptom) Scale developed originally for patients receiving allogeneic bone marrow transplantation (Kushner et al., 2008), and subsequently validated for patients with head and neck cancer undergoing radiotherapy (Gussgard, Hope, Jokstad, Tenenbaum, & Wood, 2014). Time will tell whether and how PROM instruments such as the example above may improve future patient care.

To undersigned, a discussion of which term is the most correct, PROs versus PROMs, appears to be irrational. Both terms describe different, albeit interlinked, constructs. Professionals need to be cognizant of the differences when communicating with their patients and with other stakeholders.
